# Do residents’ perceptions of being well-placed and objective presence of local amenities match? A case study in West Central Scotland, UK

**DOI:** 10.1186/1471-2458-13-454

**Published:** 2013-05-07

**Authors:** Laura Macdonald, Ade Kearns, Anne Ellaway

**Affiliations:** 1 CSO/MRC Social and Public Health Sciences Unit, 4 Lilybank Gardens, Glasgow, G12 8RZ, UK; 2 Department of Urban Studies, University of Glasgow, 25 Bute Gardens, Glasgow, G12 8RS, UK

**Keywords:** GIS, Local amenities, Built environment, Perceptions, Proximity, Neighbourhood

## Abstract

**Background:**

Recently there has been growing interest in how neighbourhood features, such as the provision of local facilities and amenities, influence residents’ health and well-being. Prior research has measured amenity provision through subjective measures (surveying residents’ perceptions) or objective (GIS mapping of distance) methods. The latter may provide a more accurate measure of physical access, but residents may not use local amenities if they do not perceive them as ‘local’. We believe both subjective and objective measures should be explored, and use West Central Scotland data to investigate correspondence between residents’ subjective assessments of how well-placed they are for everyday amenities (food stores, primary and secondary schools, libraries, pharmacies, public recreation), and objective GIS-modelled measures, and examine correspondence by various sub-groups.

**Methods:**

ArcMap was used to map the postal locations of ‘Transport, Health and Well-being 2010 Study’ respondents (n = 1760), and the six amenities, and the presence/absence of each of them within various straight-line and network buffers around respondents’ homes was recorded. SPSS was used to investigate whether objective presence of an amenity within a specified buffer was perceived by a respondent as being well-placed for that amenity. Kappa statistics were used to test agreement between measures for all respondents, and by sex, age, social class, area deprivation, car ownership, dog ownership, walking in the local area, and years lived in current home.

**Results:**

In general, there was poor agreement (Kappa <0.20) between perceptions of being well-placed for each facility and objective presence, within 800 m and 1000 m *straight-line* and *network buffers*, with the exception of pharmacies (at 1000 m *straight-line*) (Kappa: 0.21). Results varied between respondent sub-groups, with some showing better agreement than others. Amongst sub-groups, at 800 m *straight-line buffers*, the highest correspondence between subjective and objective measures was for pharmacies and primary schools, and at 1000 m, for pharmacies, primary schools and libraries. For *road network buffers* under 1000 m, agreement was generally poor.

**Conclusion:**

Respondents did not necessarily regard themselves as well-placed for specific amenities when these amenities were present within specified boundaries around their homes, with some exceptions; the picture is not clear-cut with varying findings between different amenities, buffers, and sub-groups.

## Background

In recent years there has been growing interest in the significance of neighbourhood effects on health, and how particular features of the places in which people live may influence their health and well-being [1-3]. Features of the physical environment may promote or impair health, either directly or indirectly, through the opportunities they provide for people to live healthy lives. Research has contributed to this topic by exploring the influence of accessibility to neighbourhood amenities and services [4-6]. The accessibility of specific amenities can be theorised as contributing to health and well-being in many ways; easier access to shops, leisure facilities, health services etc. offers lifestyle choices and provides cost- and time-saving benefits which frees up resources for use elsewhere [7] and may offer opportunities for physical activities (e.g. walking to destinations within easy reach) [8].

Aspects of the neighbourhood environment have been measured in different ways. Previous literature has used self-report methods to survey residents’ perceptions of neighbourhood features by asking for example, whether parks or shops were within walking distance of respondents’ homes [4,9]. Other studies have measured actual physical distance [5,10-12]. Objective measures may provide a more accurate picture of the neighbourhood physicality, but residents may not use local amenities or destinations if they are unaware of their presence, or if they do not consider them to be near or ‘local’. We therefore believe it is of interest to consider both subjective and objective measures of neighbourhood features; explore the extent to which they match and whether certain sub-groups show different relationships between the two (e.g. variation by sex, age, social class etc.). An understanding of the association between perceived and objective measures is essential, as if the former is used as a proxy for objective measures but the two bear only a weak relation then important associations may be overlooked, while others may be wrongly observed [13]. This may have implications for ‘Accessibility Planning’, that is assessment of whether people can reach key services (e.g. health care, education and food shops) within their local area; comprehensive planning should include subjective and objective measures of accessibility [14].

Most research to date has been based within regions of Australia and the US. Some of these papers reported a lack of agreement between perceived access to amenities and objective measures of access [6,8,15,16], while others found significant agreement for specific features, such as, recreation facilities [17], supermarkets [18,19], general food stores [16], schools [8,20], and libraries, [8]. Few studies have examined agreement between perceived and objective measures of local area amenities within the UK [6].

In earlier work [6] we investigated the extent to which residents’ perceived assessment of living within half a mile of a local public green space corresponded to objectively measured distance, but found agreement was only 62% and the Kappa value (i.e. an inter-rater agreement statistic which evaluates the agreement between two classifications [21]) was poor at 0.095 (Kappa is significant if it reaches >0.20) [6]. In this paper we use data from a study based in West Central Scotland, UK, to, *first,* investigate whether there is correspondence between residents’ assessments of how well-placed they are for everyday amenities (food stores, primary and secondary schools, libraries, pharmacies and public recreation or sports facilities) and GIS-modelled accessibility measures. *Secondly*, we examine agreement by various socio-demographic sub-groupings as previous literature has shown variation in perceptions of neighbourhood features by characteristics such as sex and age [22], social class and area deprivation [13,19,23]. *Finally*, we investigate whether factors which may influence the respondents’ level of experience of their neighbourhood would in turn affect the correspondence between subjective and objective assessments of accessibility, such as car ownership [24], owning a dog [25], walking within the local area [4,26], years lived at current home [13,27], and regular use of local amenities.

As most previous studies have focused on only one particular type of amenity, e.g. physical activity facilities or food retail environments, and potential variation by individual characteristics has been overlooked, our study extends this work by including a number of different amenities and explores sub-group variation. Hence, our study makes an important contribution to discussions on how to effectively measure and interpret residents’ access to local amenities.

## Methods

For this paper, we used data from one aspect of a larger study: the ‘Transport, Housing and Well-being Study’ (THAW 2010). THAW 2010 was based on THAW 1997, a study designed to examine three objectives, firstly, the statistical associations between long term morbidity and mental health and well-being on the one hand, and housing tenure and car ownership on the other (while controlling for socio-demographic and psychological characteristics); secondly, the role of housing quality, residential environment, and use of cars, in influencing illness and psychological health; and thirdly the meaning of housing tenure and car ownership in people’s daily lives [28-34]. THAW 2010 draws on respondents from similar geographical areas and uses a similar questionnaire to the previous study; for THAW 2010 a postal questionnaire, with three reminders, achieved a response rate of 38% (2092 completed questionnaires), from a random stratified sample of 5521 adults from the electoral roll in local authority areas in West Central Scotland. Survey respondents’ ages ranged from 17 to 95 years old. The survey included questions on the respondents’ mental and physical health and well-being, lifestyle, housing, neighbourhood, transport, employment, and finance. Compared to the West Central Scotland population, our study sample characteristics were broadly similar for sex and for age; 56% were female, and 65% were of working age (18 to 60 years old), compared to 52% and 62% respectively within West Central Scotland [35]. Within our sample, 85% of respondents had access to at least one car or van, while within the 2010 Scottish Household Survey, within West Central Scotland, 70% had access to a car (does not include van access) [36]. The socio-demographic characteristics of THAW 2010 were comparable to the previous THAW Study; e.g. respondents’ own social class was similar in THAW 1997 and THAW 2010 (65% and 70% in the non-manual social class groups, respectively). THAW 2010 was approved by the Ethics Committee of the Faculty of Law, Business and Social Sciences at the University of Glasgow.

In this paper we focus on six amenities: respondents were asked ‘How well-placed do you think your home is for’- ‘general food stores’, ‘primary schools’, ‘secondary schools’, ‘libraries’, ‘chemists or pharmacies’, ‘public recreation or sports facilities’. Although we did not define the term well-placed on the survey form, we expected that our respondents would interpret this term in accord with the standard definition as given in the Cambridge Dictionary as being ‘in a very convenient position’ [37]. We did not include the full set of amenities asked in our survey as some items referred to aspects of the physical environment which were difficult to measure independently e.g. ‘safe play areas’, or ‘pleasant green spaces’, or referred to resources that were fewer and further afield, e.g. ‘hospitals with a casualty department’. Respondents were asked to choose from one of four options for each resource: ‘very well-placed’, ‘fairly well-placed’, ‘not very well-placed’, or ‘not at all well-placed’. In previous research access to local amenities has been garnered using similar questions regarding how well-placed respondents’ homes are for, e.g., leisure facilities or health services [38-40]. In this study, for the purpose of analysis, and to increase the size of comparative groups, the responses were re-grouped into two categories ‘very well-placed/fairly well-placed’, and ‘not very well-placed/not at all well-placed’. For comparison, we also grouped responses into ‘very well-placed’ and ‘fairly well-placed/not very well-placed/not at all well-placed’.

Various on-line and Ordnance Survey [41] resources were used to extract the addresses of the six amenities within buffers around respondents’ postal code locations; schools’ data was obtained from the Scottish Schools Online website [42], library details from the relevant Local Authorities websites, pharmacy details from the relevant Health Board websites, and general food stores’ and public recreation/sports facilities’ data were collected from Ordnance Survey Points of Interest data (2010) [43]. Ordnance Survey Maps (including addresses and roads, paths etc.) [41] were obtained and ArcMap Version 9.3.1 was used to geocode the location of every respondent by the centroid of their unit postcode (unit postal codes are the smallest level of postal geography in the UK and typically contain around 15 address points), and the six amenities. ArcMap was used to create both Euclidean and network buffers around respondents’ home postal codes. A Euclidean buffer is a straight-line circular radius around a point, while a network buffer includes a defined distance along the pedestrian road network (i.e. roads and paths) in all possible directions away from a point, and the routes’ end points are joined to form an enclosed area. ArcMap was used to create straight-line buffers of 800 meters (m) and 1000 m around (the centroid of) each respondent postal code; the presence (or absence) of (at least one of) each of the six types of amenities within each buffer was then recorded. For network buffers, the presence of the amenities within 800 m, 1000 m, and (due to some amenities being further away on average than others) 1200 m were determined. At an average walking speed of 5 km per hour [44], 800 m relates to (approximately) a 9–10 minute walk, 1000 m a 12 minute walk, and 1200 m a 14–15 minute walk. Similar buffer sizes have been used previously [11,17,45-48].

SPSS Version 15.0 was used to investigate whether the objective presence of an amenity within a specified boundary was perceived by a respondent as being well-placed for that amenity, by cross-tabulating the objective and subjective variables (for each amenity) and calculating Kappa statistics. Kappa measures inter-rater agreement for categorical items, accounting for agreement occurring by chance (i.e. Kappa < 0.20 = poor agreement, 0.21-0.40 = fair, >0.40 = moderate to very good) [21]. Analysis was undertaken for all respondents together and also separately by sex; age tertile (17–45 years, 46–60 years, over 60 years); head of household Social Class (UK Registrar General’s Classification of Occupations [49]); and area deprivation (using the Scottish Index of Multiple Deprivation (SIMD) Income sub-domain score (based on numbers of claimants for a range of welfare benefits [50])). A look-up table was used to link the respondents’ postal codes to data zones, and for each data zone we obtained the SIMD Income score (SIMD 2009 scores divided into quintiles (Q1 = least deprived, Q5 = most deprived)). We did not undertake analysis separately by Urban/Rural scores as all respondents lived within accessible areas (93% within urban areas/accessible towns, 7% within accessible rural). We did not explore relationships between perceived and/or objective measures of access to amenities and physical activity as physical activity was not comprehensively measured within the study.

Some variables were included to measure respondents’ likely degree of experience of their local area. Thus, analysis was also undertaken by household car ownership; having a dog in the household; and whether the respondent took a walk of two miles or more in the previous year (and whether this was in or outside their local area), and years lived at current home in three groups (less than two years (<2), two to ten years, and over ten years (>10)). We included these variables to explore whether those who we assumed spent more time within their local area (i.e. non-car owners, dog owners, those who walked within the area, and those who had lived in their home for a longer period) would display better agreement.

Additionally we explored if respondents made use of supermarkets, sports facilities, and if they took children to school, and the form of transport they usually used for each. We do not know if this travel was to local amenities or those further away (data on travel to general food stores, libraries and pharmacies not gathered). We compared agreement between those who made use of the three amenities, and those who did not, and for the former we compared those who walked to the amenities and those who used other means of transport (i.e. car, taxi, public transport). Within the THAW Survey 1760 respondents had no missing values for any of the variables to be included in the analysis (with the exception of an additional eight missing responses for where respondents walked (inside and/or outside the local area), and two missing responses for time lived at current home).

## Results

### Perception of well-placed

In terms of respondents’ perceptions of being very well-placed or fairly well-placed, over 90% of the respondents believed they were for general food stores, primary schools and pharmacies, and over three quarters believed they were for recreation/sports facilities, libraries and secondary schools (see Table 1). When well-placed included the very well-placed response only, around two thirds of respondents felt well-placed for primary schools, just over half felt so for pharmacies, while less than half of respondents felt so for general food stores (46%), secondary schools (46%), libraries (38%) and public recreation/sports facilities (30%) (results not shown, available on request).

**Table 1:** Sample characteristics (N = 1760)

		**N**	**%**
**Sex**	Males	768	43.6
	Females	992	56.4
**Age tertile**	17-45	595	33.8
	46-60	635	36.1
	Over 60	530	30.1
**Social Class**	I Professional, II Managerial & Technical	988	56.1
	III Skilled non-manual	413	23.5
	III Skilled manual	202	11.5
	IV/V Partly skilled & Unskilled	157	8.9
**Area deprivation**	Quintile 1 (least deprived)	432	24.5
	Quintile 2	341	19.4
	Quintile 3	329	18.7
	Quintile 4	346	19.7
	Quintile 5 (most deprived)	312	17.7
**Car ownership**	Owner	1502	85.3
	Non-owner	258	14.7
**Dog ownership**	Owner	372	21.1
	Non-owner	1388	78.9
**Walked 2+ miles**	Walker	1366	77.6
	Non-walker	394	22.4
*Walker 2+ miles	In and out of area	1129	83.1
	Outside the area	229	16.9
**Years at current home	< 2 years	76	4.3
	2-10 years	591	33.6
	>10 years	1091	62.1
**General Food Stores**	Very/Fairly Well-Placed	1605	91.2
	Not very/Not at all Well-Placed	155	8.8
**Primary Schools**	Very/Fairly Well-Placed	1695	96.3
	Not very/Not at all Well-Placed	65	3.7
**Secondary Schools**	Very/Fairly Well-Placed	1535	87.2
	Not very/Not at all Well-Placed	225	12.8
**Libraries**	Very/Fairly Well-Placed	1510	85.8
	Not very/Not at all Well-Placed	250	14.2
**Pharmacies**	Very/Fairly Well-Placed	1656	94.1
	Not very/Not at all Well-Placed	104	5.9
**Public Sport/Recreation Facilities**	Very/Fairly Well-Placed	1371	77.9
	Not very/Not at all Well-Placed	389	22.1

*8 missing, **2 missing.

### Measured distance

Using straight-line buffers, the majority of respondents lived within 800 m of a general food store (92%) and of a primary school (86%), while over 70% of respondents lived within 800 m of a pharmacy and of a recreation/sports facility (see Table 2). Less than half of the respondents lived within an 800 m buffer of a secondary school (38%) or a library (45%), but this rose to 50% and 60%, respectively, at 1000 m.

**Table 2:** Number, percentage, of respondents within 800 m, 1000 m straight-line buffers, and 800 m, 1000 m network buffers, of amenities

	**800 m straight-line buffers**		**1000 m straight-line buffers**		**800 m network buffers**		**1000 m network buffers**	
	**N**	**%**	**N**	**%**	**N**	**%**	**N**	**%**
**General Food Stores**	1624	92.3	1680	95.5	1227	69.7	1448	82.3
**Primary Schools**	1523	86.5	1643	93.4	942	53.5	1199	68.1
**Secondary Schools**	675	38.4	891	50.6	277	15.7	419	23.8
**Libraries**	793	45.1	1062	60.3	317	18.0	506	28.8
**Pharmacies**	1267	72.0	1445	82.1	795	45.2	1019	57.9
**Public Recreation or Sports Facilities**	1335	75.9	1525	86.6	557	31.6	802	45.6

In terms of path/road network buffers, over half of the respondents (69%) lived within 800 m of a general food store or a primary school (53%), rising to 82% and 68%, respectively, within 1000 m. A minority of respondents lived within 1000 m of a secondary school, library or a recreation/sports facility (23%, 28%, and 45% respectively); while less than three in five lived within 1000 m of a pharmacy (see Table 2).

### Agreement - all respondents

There was poor Kappa agreement (<0.20) between perceptions of being very well-placed or fairly well-placed for each facility and availability within a distance of an 800 m *straight-line buffer*, while agreement was fair for perceptions and a 1000 m buffer for pharmacies only (Kappa = 0.21) (see Table 3). When comparing perceptions and objectively measured *network buffers*, of 800 m, 1000 m and 1200 m, agreement was poor for all facilities (<0.20) (results for 1200 m not shown, available on request). Respondents were thus no more likely to regard themselves as well-placed for specific amenities when those amenities were present within specified buffers around their homes, as when not (except pharmacies).

**Table 3:** Agreement (Kappa) between perceptions of well-placed and measured distance to amenities

	**800 m straight-line buffers**	**1000 m straight-line buffers**	**800 m network buffers**	**1000 m network buffers**
	**Kappa Value**	**Kappa Value**	**Kappa Value**	**Kappa Value**
**General Food Stores**	0.127	0.126	0.091	0.138
**Primary Schools**	0.149	0.181	0.041	0.080
**Secondary Schools**	0.088	0.140	0.028	0.052
**Libraries**	0.133	0.186	0.051	0.082
**Pharmacies**	0.144	0.206*	0.075	0.106
**Public Recreation or Sports Facilities**	0.118	0.139	0.015	0.048

*Fair Kappa value >0.20 (all others poor).

### Straight-line buffers

There was no difference in agreement, between perceptions of being well-placed for each of the six amenities and 800 m straight-line buffers, when groups were analysed separately by sex, age, dog ownership, and area walked in (all Kappa values poor (<0.20)) (see Table 4). Fair agreement (Kappa >0.20) was shown for the following amenities and sub-groups: food stores (more deprived areas (Q3 & Q4), non-walkers, <2 years in current home); primary schools (IIIm, Q2 & Q4 area deprivation, non-car owners, <2 years in current home); libraries (IV/V and non-car owners); pharmacies (IV/V, Q3 area deprivation, non-car-owners, non-walkers, <2 years in current home); and recreation/sports facilities (Q3 area deprivation). Therefore, primary schools and pharmacies showed the most fair Kappa values amongst sub-groups (5 out of 24), while fair agreement was most often achieved by those in Q3 are deprivation, non-car owner and <2 years in current home sub-groups (three amenities each).

**Table 4:** Agreement between perceptions of well-placed and straight-line 800 m buffer to amenities by sub-groups

		**Food stores**	**Primary schools**	**Secondary schools**	**Libraries**	**Pharmacies**	**Sport/Recreation**
		**Kappa**	**Kappa**	**Kappa**	**Kappa**	**Kappa**	**Kappa**
**Sex**	Males (768)	0.080	0.124	0.090	0.158	0.158	0.090
	Females (992)	0.161	0.165	0.086	0.112	0.133	0.140
**Age tertile**	17-45 (595)	0.108	0.169	0.077	0.126	0.122	0.113
	46-60 (635)	0.122	0.117	0.090	0.153	0.146	0.098
	Over 60 (530)	0.142	0.176	0.092	0.115	0.160	0.131
**Social Class**	I/II/III n-m (1401)	0.134	0.151	0.077	0.119	0.128	0.124
	III manual (202)	0.065	0.240*	0.170	0.145	0.144	0.123
	IV/V (157)	0.180	0.066	0.097	0.237*	0.347*	0.095
**Area deprivation**	Quintile 1 (432)	0.051	0.074	0.037	0.104	0.094	0.049
	Quintile 2 (341)	0.081	0.216*	0.104	0.103	0.118	0.139
	Quintile 3 (329)	0.296*	0.162	0.127	0.164	0.277*	0.229*
	Quintile 4 (346)	0.213*	0.213*	0.075	0.106	0.078	0.048
	Quintile 5 (312)	0.069	0.197	0.131	0.198	0.168	0.174
**Car ownership**	Owner (1502)	0.140	0.132	0.082	0.115	0.134	0.111
	Non-owner (258)	0.078	0.269*	0.136	0.248*	0.241*	0.178
**Dog ownership**	Owner (372)	0.120	0.162	0.067	0.133	0.171	0.159
	Non-owner (1388)	0.127	0.145	0.093	0.131	0.135	0.107
**Walked 2+ miles**	Walker (1366)	0.083	0.143	0.092	0.122	0.126	0.116
	Non-walker (394)	0.228*	0.167	0.070	0.159	0.217*	0.142
**Area walked**	In & out of area (1129)	0.108	0.154	0.094	0.137	0.152	0.111
	Outside the area (229)	−0.060	0.047	0.089	0.042	−0.018	0.151
**Years at current home**	< 2 years (76)	0.303*	0.288*	0.156	0.115	0.286*	0.099
	2-10 years (591)	0.077	0.101	0.079	0.127	0.186	0.070
	> 10 years (1091)	0.132	0.158	0.087	0.139	0.117	0.136

*Fair Kappa Value.

When extending to 1000 m, fair agreement was more often achieved than at an 800 m distance. The majority of Kappa values which were fair for 800 m remained (see Table 5), in addition, fair agreement was seen for primary schools (for over 60 s, Q3 area deprivation, dog owners, walkers in the local area, >10 years in current home sub-groups); secondary schools (social class IIIm, Q5 area deprivation, non-car owners, <2 years in current home); libraries (males, 46–60 year olds, Q3 & Q5 area deprivation, non-walkers, <2 years in current home); and pharmacies (males, 46–60 year olds, over 60 s, social class IIIm, dog owners, local area walkers, 2–10 years in current home). Therefore, pharmacies, primary schools and libraries, showed the highest occurrence of fair/moderate Kappa values, and those in the Q3 area deprivation sub group, and those who resided less than 2 years in their current home showed the highest number of fair values (five and four amenities respectively).

**Table 5:** Agreement between perceptions of well-placed and straight-line 1000 m buffer to amenities by sub-groups

		**Food stores**	**Primary schools**	**Secondary schools**	**Libraries**	**Pharmacies**	**Sport/Recreation**
		**Kappa**	**Kappa**	**Kappa**	**Kappa**	**Kappa**	**Kappa**
**Sex**	Males (768)	0.081	0.157	0.141	0.219*	0.218*	0.127
	Females (992)	0.155	0.197	0.138	0.159	0.197	0.147
**Age tertile**	17-45 (595)	0.065	0.162	0.129	0.176	0.156	0.164
	46-60 (635)	0.167	0.188	0.140	0.207*	0.241*	0.102
	Over 60 (530)	0.118	0.201*	0.145	0.169	0.206*	0.147
**Social Class**	I/II/III n-m (1401)	0.148	0.196	0.125	0.183	0.195	0.148
	III manual (202)	0.009	0.220*	0.233*	0.144	0.210*	0.121
	IV/V (157)	0.134	0.069	0.168	0.255*	0.320*	0.103
**Area deprivation**	Quintile 1 (432)	0.072	0.086	0.049	0.130	0.133	0.076
	Quintile 2 (341)	0.041	0.388*	0.168	0.174	0.185	0.192
	Quintile 3 (329)	0.316*	0.208*	0.190	0.233*	0.348*	0.276*
	Quintile 4 (346)	0.167	0.304*	0.114	0.184	0.184	0.077
	Quintile 5 (312)	0.033	0.144	0.274*	0.230*	0.166	0.109
**Car ownership**	Owner (1502)	0.145	0.183	0.130	0.160	0.199	0.139
	Non-owner (258)	0.052	0.181	0.229*	0.346*	0.275*	0.154
**Dog ownership**	Owner (372)	0.126	0.212*	0.157	0.175	0.235*	0.165
	Non-owner (1388)	0.123	0.170	0.134	0.187	0.197	0.131
**Walked 2+ miles**	Walker (1366)	0.105	0.180	0.132	0.171	0.188	0.134
	Non-walker (394)	0.167	0.181	0.167	0.217*	0.255*	0.153
**Area walked**	In & out of area (1129)	0.127	0.204*	0.137	0.185	0.209*	0.145
	Outside the area (229)	−0.033	−0.028	0.118	0.115	0.036	0.076
**Years at current home**	< 2 years (76)	0.253*	0.139	0.227*	0.216*	0.424**	0.114
	2-10 years (591)	0.098	0.094	0.152	0.170	0.221*	0.095
	> 10 years (1091)	0.126	0.230*	0.128	0.194	0.186	0.152

*Fair Kappa Value, **Moderate Kappa value.

### Road network buffers

When using road network buffers at thresholds of 800 m and 1000 m all Kappa values were poor for all the facilities and sub-groups, with the exception of <2 years in current home and food stores at 1000 m. When using 1200 m network buffers, some sub-groups displayed fair agreement for food stores (Q3 area deprivation, non-walkers, <2 years in current home); primary schools (social class IIIm, Q3 area deprivation); pharmacies (Q3 area deprivation, non-walkers, IV/V, <2 years in current home); and recreation/ sports facilities (Q3 area deprivation) (results not shown, available on request).

### Use of and means of travel to supermarkets, sports facilities, and schools

Only 1.6% of the sample reported that they did not go to supermarkets, 24.9% did not go to sports facilities and 57.6% did not take children to school. Around 20% of respondents walked to supermarkets, just over 20% walked to sports facilities, while around 40% walked their children to school. There was no difference in agreement between perceptions of being well-placed and GIS modelled distance for those who made use of, and those who did not make use of, supermarkets, sports facilities or schools. Moreover, the form of transport to each of the three amenities did not affect agreement (i.e. walking versus cars/vans, taxis or public transport) (true for straight-line or network buffers) (not shown, available on request).

### Sensitivity analysis

When analysing data using responses grouped into ‘very well-placed’ versus ‘fairly well-placed/not very well-placed/not at all well-placed’, fair agreement was seen for pharmacies within 800 m *straight-line buffers*, and 800 m and 1000 m *network buffers*, while fair agreement was shown for libraries within 800 m and 1000 m *straight-line buffers*.

For 800 m *straight-line* buffers most sub-groups showed fair Kappa values for libraries (23) and pharmacies (22) (17 and 10 subgroups respectively at 1000 m). Social class group IV/V showed the highest number of fair Kappa values, for primary and secondary schools, libraries and pharmacies within 800 m *straight-line* buffers (and 800 m and 1000 m *network buffers*). For 800 m *network buffers*, 23 out of 24 sub-groups exhibited fair agreement for pharmacies (21 at 1000 m), while nine sub-groups did so for libraries (15 at 1000 m) (not shown, available on request).

## Discussion

Our results showed that residents’ perceptions of being well-placed for everyday amenities, such as food stores, schools, libraries and sports/recreation facilities, was poorly matched with geographically modelled access (or simply put, respondents were more likely to perceive an amenity as closer or farther than it physically was); the only exception was for pharmacies where agreement was fair (for 1000 m straight-line buffers).

Some respondent sub-groups appeared to show better agreement, i.e. males, older age groups, lower social class groups, those in Q3 of area deprivation, non-car owners, dog owners, those who did not take a walk of more than 2 miles in the past year. Among those who *did* report having taken such a walk, those who did this in their local area had a better level of agreement (than those who walked outside the local area), as did those who had lived in their current home for less than 2 years. Thus, there was some evidence that those who may have greater experience of their local neighbourhood exhibited greater correspondence between subjective and objective assessments of accessibility. Interestingly, this may include those who have lived in the area less rather than more time, possibly as a result of exploring the local area in early months and years of residence.

Results varied according to the categorisation of well-placed, the resource examined and the objective distance employed. We found variation between the 800 m and 1000 m buffers, with the latter more likely to display fair agreement; and observed variation between the straight-line buffer and network buffer analyses, which may be due to the latter covering smaller geographical areas. However, it is conceivable that when respondents considered well-placed they were not considering specific road/path network journeys [6]. When analysing data using responses grouped into ‘very well-placed’ versus ‘fairly well-placed/not very well-placed/not at all well-placed’, results differed; a greater number of sub-groups displayed fair kappa values, for libraries and pharmacies in particular, and more so within the 800 m buffer than the 1000 m buffer (as seen in the sensitivity analysis).

Of the respondents who displayed poor agreement for food stores, primary schools and sports/recreation facilities, there were similar proportions of those who considered their homes well-placed but yet had no amenities within 1000 m, and of those who did not consider themselves well-placed but had an amenity within 1000 m. In contrast, for secondary schools, libraries and pharmacies, of those with a mismatch, the majority thought they were well-placed but had no amenities nearby; resulting perhaps from these amenities displaying the lowest representation across the buffers, and lowest overall density.

It is difficult to theorise why there would be fair agreement between perceptions of being well-placed for a pharmacy and objective measures, as pharmacies were not more abundant, and the frequency of trips to other local amenities, such as food stores, in general, may be higher [51]. It may be that pharmacies are more visible within the local community because of the important part pharmacists play in primary health care; pharmacies are an accessible health resource for everyone as they often have longer opening hours than GP surgeries, appointments are not needed, and they provide a range of services, e.g. dispensing of drugs, health and lifestyle advice, signposting to other services, support for self-care, medicine use reviews, health checks, access to emergency contraception, and addiction services [52]. It is also the case that the quantity of drugs prescribed in Scotland has risen by a third in a recent years (2004–11), so that the use of pharmacies is increasing; indeed, it is reported that 60% of the population of Scotland receive at least one prescription in any three month period [53].

Prior studies have compared objective and subjective neighbourhood features by socio-demographic characteristics, with varying results. Research in Australia found that males were more likely to show mismatch between perceived and objective access to supermarkets, but there were no significant sex or age differences for access to sports fields, or libraries [22]. In our sample, males showed better match for libraries and pharmacies than females. A study within neighbourhoods in Glasgow, UK, showed that females were much more likely to do food shopping in the local area [54], but in our study females were not more likely than males to exhibit better match for food stores. In the present study, the youngest age group was more likely to display mismatch, while older groups showed fair agreement for certain amenities; over 90% of the over 60 s were retired so may spend more time at/around home, and thus have better knowledge of local amenities.

In our study there was no clear pattern of agreement by Social Class or income but non-manual classes and those within more affluent areas showed poor agreement for all amenities. Previous research undertaken in West Central Scotland, found that those with lower incomes were more likely to shop for basic food items locally [55] and may therefore be more likely to use other local facilities. Furthermore, in the present study, non-manual classes and Q1 (the least deprived areas) had the greatest car access so would be able to go further afield for activities, such as food shopping. Indeed those without cars did show fair agreement for schools, libraries and pharmacies while car owners, and those who in general used cars to travel to supermarkets, sports facilities and schools, showed mismatch for all amenities, perhaps echoing findings from other studies that perceptions of travel time may be influenced by car ownership [24,56].

Those respondents who had a dog in their household showed better agreement for primary schools and for pharmacies than those without a dog (at 1000 m). Owning a dog may encourage people to go for more walks in their neighbourhood [25]. Among those who had walked at least two miles in the local area, agreement was fair for primary schools and pharmacies, while those who tended to take walks further afield showed poor agreement for all amenities, this is in line with previous studies showing that people who engaged in leisure-time walking within their own neighbourhood were more aware of neighbourhood features [4,26]. We found that those who had lived in their current home for less than 2 years showed better agreement for four of the six amenities studied; this contrasts to previous studies which found that newcomers took a longer time to orientate themselves with neighbourhood features [13,27].

Overall, in this study, there was correspondence between respondents’ perceptions and the GIS measures for some but not all the amenities. The lack of agreement here, and similarly in other literature [6,15,16], could reflect that people generally inaccurately estimate how far or near a destination is [17,57]. Previous work has shown that various factors could influence ‘cognised’ distance to environmental built attributes; e.g., a route with more intersections and barriers (i.e. buildings and trees) could lead to overestimation of recalled distance [58,59], while an increase in physical effort, e.g. going uphill, could also lead to overestimation [60]. A respondent may appear to be objectively well-placed but access may be limited by a route which is deemed unsafe or undesirable leading to overestimation of perceived distance [23], while subjective distance to a resource could be underestimated if the respondent equates being well-placed with attractiveness of the destination [24,61].

Respondents’ self-reports of being well-placed could be influenced by the fact they do not use local facilities regularly or at all. Respondents may not rely on their home location for amenities but use other non home locations such as areas around work, study, child’s school or transport hub [62]. For some people the area around home may be of little relevance to daily life, and this could lead to inaccurate perceptions of proximity [24].

Our study displayed a number of limitations. Our analysis is based on a general question, we do not know the distance respondents consider well-placed, and do not know whether the respondent regarded the question as relating to walking or driving. Nonetheless, we do not believe that using a more specific question would have provided more accurate responses, such as “*is your home a 10 minute walk from a library?”*, as walking could be affected by walking speed, mobility etc. Moreover, we do not know if respondents’ answers related to ‘spatial’ and/or ‘non-spatial’ factors e.g. amenity prices, quality of service, range of goods on sale, or opening hours [38]. We found variation in results according to how responses were categorised (i.e. combining ‘very well-placed’ and ‘fairly well-placed’ or including the latter only) which emphasises the difficulty of associating respondents’ ideas of well-placed with objective distance. We recognise that being well-placed does not necessarily equate with neighbourhood satisfaction [63], and that residential proximity may not be imperative in influencing usage of local amenities; the quality of a resource may be of greater importance but we do not have this data, nor do we possess data on which amenities respondents use. People may not use adjacent amenities to their home as they may be unsuitable, for example a study in Glasgow found that while participants agreed that objective access to green space was important, it was not the only factor which influenced usage. They discussed personal reasons for not using green space, e.g. being fearful of potential anti-social behaviour from young people present within a park; or simply not wanting to associate with others from the local community [64]. These barriers could influence the usage of other local resources. We did not explore links between perceptions and objective measures of the built environment and actual physical activity behaviours, e.g., utilitarian walking, as we do not have this data.

Our approach comparing objective and subjective measures of accessibility raises questions about the role of perceived convenience in local amenity use (the latter having potential benefits, for example, in terms of health behaviours and community vitality). Past research has shown that frequency of amenity patronage is influenced by perceived distance, and that choice of amenity/outlet is influenced by perceived locational convenience [65]. However, other research has drawn a distinction between different types of services, with locational convenience being more important for customer loyalty (when satisfaction is low) in the case of standardised, less personal services, and less influential upon customer choice in the case of less standardised, more personal services [66]. Our findings of a greater correspondence between perceived and objective convenience in the case of pharmacies, schools and libraries, therefore raises questions as to whether this is reflected in higher usage of these amenities and/or is reflective of a view of these amenities as being of a more or less personalised nature. These are two issues to be pursued in further research.

The findings also have implications for debates around ‘Accessibility Planning’ and policy formation. Accessibility Planning assesses whether people are able to reach the key services they need/are important to them (i.e. health care, education and food shops), and contends that comprehensive planning should include considerations of not only objectively modelled distance to key services, but also residents concerns about accessibility [14] e.g. how well placed do they feel. In this way barriers to access can be investigated [38] whether these relate to a lack of physical access to services, or residents simply not knowing what is available. Our study also has implications for the development of public health interventions which aim to increase active travel (walking, cycling) such as providing walking maps to increase awareness of proximity to community resources [67].

## Conclusion

Despite its limitations we believe a major strength of this study is the contribution to the limited UK literature on comparisons of subjective and objective access to neighbourhood amenities, and inclusion of a variety of amenities covering health, education and leisure. As in previous studies [4,13,19,22,23,26] our study highlights the importance of considering that agreement between perceptions and reality of proximity to amenities may vary when comparing certain population subgroups and socio-demographic characteristics.

## Competing interests

The authors declare that they have no competing interests.

## Authors’ contributions

All authors contributed to the study design. LM undertook collection of geographical data, mapping and data analysis. All authors contributed to the interpretation of the data. LM wrote the first draft of the paper and AK and AE read the draft and provided critical comments. All authors read and approved the final draft of the paper.

## Pre-publication history

The pre-publication history for this paper can be accessed here:

http://www.biomedcentral.com/1471-2458/13/454/prepub

## References

[B1] Ellaway A Macintyre S Brown T, McLafferty S, Moon G Neighbourhoods and health The Companion to Health and Medical Geography 2009 Oxford: Blackwell Publishing 399 417

[B2] Macintyre S Ellaway A Kawachi I, Berkman L Neighbourhoods and health: an overview Neighbourhoods and health 2003 Oxford: Oxford University Press 20 42

[B3] Macintyre S Ellaway A Cummins S Place effects on health: how can we conceptualise, operationalise and measure them? Soc Sci Med 2002 55 125 139 10.1016/S0277-9536(01)00214-3 12137182

[B4] Humpel N Owen N Leslie E Environmental factors associated with Adults’ participation in physical activity a review Am J Prev Med 2002 22 188 189 10.1016/S0749-3797(01)00426-3 11897464

[B5] Macdonald L Ellaway A Macintyre S Ball K Is proximity to a food retail store associated with diet and BMI in Glasgow, Scotland? BMC Publ Health 2011 11 464 10.1186/1471-2458-11-464 PMC3128028 21663674

[B6] Macintyre S Macdonald L Ellaway A Lack of agreement between measured and self-reported distance from public green parks in Glasgow, Scotland Int J Behav Nutr Phys Act 2008 5 10.1186/1479-5868-5-26 PMC2396666 18454876

[B7] Pearce J Witten K Bartie P Neighbourhoods and health: a GIS approach to measuring community resource accessibility J Epidemiol Community Health 2006 60 389 395 10.1136/jech.2005.043281 16614327 PMC2563982

[B8] Tilt J Unfried T Roca B Using objective and subjective measures of neighborhood greenness and accessible destinations for understanding walking trips and BMI in Seattle, Washington Am J Heal Promot 2007 21 371 379 10.4278/0890-1171-21.4s.371 17465183

[B9] Ball K Bauman A Leslie E Owen N Perceived environmental and social influences on walking for exercise in Australian adults Prev Med 2001 33 434 440 10.1006/pmed.2001.0912 11676585

[B10] Coombes E Jones A Hillsdon M The relationship of physical activity and overweight to objectively measured green space accessibility and use Soc Sci Med 2010 70 816 822 10.1016/j.socscimed.2009.11.020 20060635 PMC3759315

[B11] Lotfi S Koohsari M Measuring objective accessibility to neighborhood facilities in the city (A case study: Zone 6 in Tehran, Iran) Cities 2009 26 133 140 10.1016/j.cities.2009.02.006

[B12] Macintyre S Macdonald L Ellaway A Do poorer people have poorer access to local resources and facilities? The distribution of local resources by area deprivation in Glasgow, Scotland Soc Sci Med 2008 67 900 914 10.1016/j.socscimed.2008.05.029 18599170 PMC2570170

[B13] Ball K Jeffery R Crawford D Roberts R Salmon J Timperio A Mismatch between perceived and objective measures of physical activity environments Prev Med 2008 47 294 298 10.1016/j.ypmed.2008.05.001 18544463

[B14] Department of Health Accessibility planning – an introduction for the NHS 2004 London: Department of Health, Health Inequalities Unit

[B15] Leslie E Sugiyama T Ierodiaconou D Kremer P Perceived and objectively measured greenness of neighbourhoods: Are they measuring the same thing? Landscape Urban Plann 2010 95 28 33 10.1016/j.landurbplan.2009.11.002

[B16] McGinn AP Evenson K Herring AH Huston SL Rodriguez DA Exploring associations between physical activity and perceived and objective measures of the built environment J Urban Health 2007 84 162 184 10.1007/s11524-006-9136-4 17273926 PMC2231636

[B17] Kirtland K Porter D Addy C Neet M Williams J Sharpe P Neff L Kimsey C Jr Ainsworth B Environmental measures of physical activity supports perception versus reality Am J Prev Med 2003 24 323 331 10.1016/S0749-3797(03)00021-7 12726870

[B18] Moore L Diez Roux A Brines S Comparing perception-based and geographic information system (GIS)-based characterizations of the local food environment J Urban Health 2008 85 206 216 10.1007/s11524-008-9259-x 18247121 PMC2430123

[B19] Gebel K Bauman A Owen N Correlates of Non-concordance between perceived and objective measures of walkability Ann Behav Med 2009 37 228 238 10.1007/s12160-009-9098-3 19396503

[B20] Donaldson K Streeter M Measured versus reported distances in the American housing survey 2011 Washington, DC: United States Census Bureau

[B21] Altman D Practical statistics for medical research 1993 London: Chapman and Hall

[B22] McCormack G Cerin E Leslie E Du Toit L Owen N Objective versus perceived walking distances to destinations Environ Behav 2008 40 401 425

[B23] Williams LK Ball K Thornton LE Crawford D Is the objective food environment associated with perceptions of the food environment? Public Health Nutr 2012 15 291 298 10.1017/S1368980011001947 21835079

[B24] Briggs R Downs RM, Stea D Urban distance cognition Image and environment 1976 Chicago: Aldine 361 388

[B25] Wood L Giles-Corti B Bulsara M The pet connection: pets as a conduit for social capital? Soc Sci Med 2005 61 1159 1173 10.1016/j.socscimed.2005.01.017 15970228

[B26] McCormack G Giles-Corti B Lange A Smith T Martin K Pikora TJ An update of recent evidence of the relationship between objective and self-report measures of the physical environment and physical activity behaviours J Sci Med Sport 2004 7 81 92 15214606 10.1016/s1440-2440(04)80282-2

[B27] Gatrell A Distance and space 1983 New York: Oxford University Press

[B28] Kearns A Ellaway A Macintyre S Hiscock R The THAW report: findings of a study of transport, housing and wellbeing in the West of Scotland 2000 Glasgow: Department of Urban Studies, University of Glasgow and MRC Social and Public Health Sciences Unit

[B29] Macintyre S Hiscock R Kearns A Ellaway A Housing tenure and car access: further exploration of the nature of their relationships with health in a UK setting J Epidemiol Community Health 2001 55 330 331 10.1136/jech.55.5.330 11297651 PMC1731881

[B30] Hiscock R Macintyre S Ellaway A Kearns A Residents and residence: Factors predicting the health disadvantage of social renters compared to owner-occupiers J Soc Issues 2003 59 527 546 10.1111/1540-4560.00076

[B31] Ellaway A McKay L Macintyre S Kearns A Hiscock R Are social comparisons of homes and cars related to psychosocial health? Int J Epidemiol 2004 33 1065 1071 10.1093/ije/dyh197 15256528

[B32] Hiscock R Ellaway A Kearns A Macintyre S Transport, housing and well-being in West Central Scotland, Working Paper 69 1999 Glasgow: MRC Social & Public Health Sciences Unit

[B33] Macintyre S Ellaway A Hiscock R Kearns A Der G McKay L What features of the home and area might help to explain observed relationships between housing tenure and health? evidence from the west of Scotland Health & Place 2003 9 207 218 10.1016/S1353-8292(02)00040-0 12810328

[B34] Ellaway A Macintyre S Hiscock R Kearns A In the driving seat: psychosocial benefits from private motor vehicle transport compared to public transport Transport Res F: Traffic Psychol Behav 2003 6 217 231 10.1016/S1369-8478(03)00027-5

[B35] GROS Mid-2010 population estimates Scotland http://www.gro-scotland.gov.uk/statistics/theme/population/estimates/mid-year/2010/tables.html

[B36] Scottish household survey - annual report 2009/2010 - LA tables - excel workbook http://www.scotland.gov.uk/Topics/Statistics/16002/LA0910Excel

[B37] Definition of well-placed http://dictionary.cambridge.org/dictionary/learner-english/well-placed

[B38] Fone D Christie S Lester N Comparison of perceived and modelled geographical access to accident and emergency departments: a cross-sectional analysis from the Caerphilly Health and Social Needs Study Int J Health Geogr 2006 5 10.1186/1476-072X-5-16 PMC1526712 16613601

[B39] Harrison R Gemmell I Heller R The population effect of crime and neighbourhood on physical activity: an analysis of 15 461 adults J Epidemiol Community Health 2007 61 34 39 10.1136/jech.2006.048389 17183012 PMC2465585

[B40] Poortinga W Dunstan F Fone D Perceptions of the neighbourhood environment and self rated health: a multilevel analysis of the Caerphilly Health and Social Needs Study BMC Public Health 2007 7 10.1186/1471-2458-7-285 PMC2100049 17925028

[B41] Ordnance Survey http://www.ordnancesurvey.co.uk/oswebsite/

[B42] Learning and teaching Scotland- Scottish schools online http://www.ltscotland.org.uk/scottishschoolsonline/index.asp

[B43] Ordnance survey points of interest http://www.ordnancesurvey.co.uk/oswebsite/products/points-of-interest/index.html

[B44] Ogilvie D Lamb KE Ferguson N Ellaway A Recreational physical activity facilities within walking and cycling distance: Sociospatial patterning of access in Scotland Health & Place 2011 17 1015 1022 10.1016/j.healthplace.2011.07.003 21816656

[B45] Apparicio P Cloutier M Shearmur R The case of Montréal’s missing food deserts: evaluation of accessibility to food supermarkets Int J Health Geogr 2007 6 10.1186/1476-072X-6-4 PMC1803774 17295912

[B46] Li F Fisher KJ Brownson RC Bosworth M Multilevel modelling of built environment characteristics related to neighbourhood walking activity in older adults J Epidemiol Community Health 2005 59 558 564 10.1136/jech.2004.028399 15965138 PMC1757084

[B47] Lin L Moudon AV Objective versus subjective measures of the built environment, which are most effective in capturing associations with walking? Health & Place 2010 16 339 348 10.1016/j.healthplace.2009.11.002 20004130

[B48] Smoyer-Tomic K Spence J Amrhein C Food deserts in the prairies? supermarket accessibility and neighborhood need in Edmonton, Canada Prof Geographer 2006 58 307 326 10.1111/j.1467-9272.2006.00570.x

[B49] Office of Population Censuses and Surveys OPCS standard occupational classification 1991 London: HMSO

[B50] Scottish index of multiple deprivation http://www.scotland.gov.uk/Topics/Statistics/SIMD/Background-Data-2009

[B51] Gebel K Bauman A Sugiyama T Owen N Mismatch between perceived and objectively assessed neighborhood walkability attributes: Prospective relationships with walking and weight gain Health & Place 2011 17 519 524 10.1016/j.healthplace.2010.12.008 21233002

[B52] NHS 24. How can the pharmacy help me? http://www.nhs24.com/FindLocal/Pharmacies

[B53] Scotland AG Prescribing in general practice in Scotland 2013 Edinburgh: Audit Scotland

[B54] Macintyre S Ellaway A Social and local variations in the use of urban neighbourhoods: a case study in Glasgow, Scotland Health Place 1998 4 91 94 10.1016/S1353-8292(97)00030-0 10671014

[B55] Ellaway A Macintyre S Shopping for food in socially contrasting localities Br Food J 2000 102 52 69 10.1108/00070700010310632

[B56] Crompton A Brown F Distance Estimation in a Small-Scale Environment Environ Behav 2006 38 656 666 10.1177/0013916505281571

[B57] Canter D Tagg SK Distance estimation in cities Environ Behav 1975 7 59 80 10.1177/001391657500700102

[B58] Cohen R Weatherford DL Effects of route traveled on the distance estimates of children and adults J Exp Child Psychol 1980 29 403 412 10.1016/0022-0965(80)90103-4 7373211

[B59] Sadalla EK Magel SG The perception of traversed distance Environ Behav 1980 12 65 69 10.1177/0013916580121005

[B60] Okabe A Aoki K Hamamoto W Distance and direction judgement in a large-scale natural environment: Effects of a slope and winding trail Environ Behav 1986 18 755 772 10.1177/0013916586186004

[B61] Walmsley DJ Jenkins JM Cognitive distance: a neglected issue in travel behavior J Travel Res 1992 31 24 29 10.1177/004728759203100106

[B62] Hurvitz PM Moudon AV Home versus nonhome neighborhood quantifying differences in exposure to the built environment Am J Prev Med 2012 42 411 417 10.1016/j.amepre.2011.11.015 22424255 PMC3318915

[B63] Hawthorne TL Kwan MP Using GIS and perceived distance to understand the unequal geographies of healthcare in lower-income urban neighbourhoods Geogr J 2012 178 18 30 10.1111/j.1475-4959.2011.00411.x 22400154

[B64] Seaman P Jones R Ellaway A It's not just about the park, it's about integration too: why people choose to use or not use urban greenspaces Int J Behav Nutr Phys Act 2010 7 10.1186/1479-5868-7-78 PMC2978120 21029448

[B65] Jones MA Mothersbaugh DL Beatty SE The effects of locational convenience on customer repurchase intentions across service types J Serv Mark 2003 17 701 712 10.1108/08876040310501250

[B66] Spatial consumption decision-making: Six studies of restaurant choice http://sunzi.lib.hku.hk/ER/detail/hkul/2993439

[B67] McNeill L Emmons K GIS walking maps to promote physical activity in low-income public housing communities: a qualitative examination Prev Chronic Dis 2012 9 PMC3277385 22172184

